# Personal Beauty

**Published:** 1874-04

**Authors:** P. H. Hayes


### PERSONAL BEAUTY.

P. H. HAYES, M. D.

To be fat is the royal style for beauty in Turkey. A beautiful woman, in the eyes of a Turkish lord, must be a load for a camel. But their women are not born to this estate of fatness, it is put on by stuffing with butter and pounded rose leaves and idleness, and semi-imbecility. An English lady who was often admitted to the harem of the late Sultan, declares that she there saw five hundred of these beauties, (?) and not one truly beautiful woman "in the whole vast accumulation of rose leaves and butter." These women have all a pale and sallow look in their faces, and a grossness of ensemble which may answer well to the Moslem's idea of a sensual paradise, but can excite only aversion in a refined mind.

I have brought forward this illustration of what diet and inertia of mind and body can do, to point a moral of beauty for my fair country women. I need not stop to argue for the charm of the presence of a really beautiful woman, a woman in whom form and feature and color and soul and spirit conspire to set a spell upon our souls. By natural sagacity, women themselves know the value of beauty, strive to promote it in themselves, and bow to it even in their sisters, by envy, or by admiration. Madame de Stael would give half her knowledge for beauty.

The young girls of the Turkish royalty have natural beauty. Their diet destroys it. And think you, who read my ramble, that multitudes of our girls are not spoiling their beauty by spoiling their digestion and interrupting the work of the liver? What else can be expected from a breakfast of hot bread, hot cakes with the grease burned in, butter and sugar and strong coffee? The mid-day meal is too often but a light lunch, to stay the stomach and make it furious for the late-in-the-day dinner. And now the half-famished girl sits down and sates a keen appetite with peppered soups, fish, meats, game, tarts, pies, cake, puddings, ice cream, with the addition perhaps of tea, coffee, or wine. A perfect digestion of this melange is impossible. Witness the flushed and burning complexion, and guess at the fever within, and the task of poor stomach and liver. Add now to this, that which so often occurs with those who spend their evenings out—a bed-time lunch—and do you wonder that the firm texture of the body gives way to flabbiness, and roundness and contour yield to leanness, and that by the fifth quinquenium, the beauty's looking-glass says to her unwilling ear, through her unwilling eye, "we all do fall as a leaf."

No young woman who would enjoy beauty can afford to impair her digestion. The stomach is the foundation, the nourisher, the renovator of life. It is the organ which makes the blood and the blood is the *life;* from the blood comes color, warmth, inspiration, courage. A weak digestion makes a poor blood, a pale face, flabby flesh, lustreless eyes. It is the sure work of coffee to stimulate the brain and weaken the stomach, and the one is the counterpart of the other. Mrs. Blanque takes her prematurely fading daughter to a physician of brains and wants to know why Belle is thin and losing color and spirit. Why, Madam, you are starving your daughter! Indignant mother answered tartly, that her daughter has every luxury and delicacy that money can buy or the art of the cook supply. Does she take tea and coffee? Yes, and we have tried wine. Any nuts and candies? Not a *great many!* But, Madam, your daughter is *starving* upon luxuries and delicacies. Her stomach is spoiled by them, her blood-making organs fail, and her body languishes. The body lives on blood, and the stomach must make it from the food, if not the body starves. Give your daughter bread and butter, bread and milk, fruit, a little fresh beef or mutton for dinner, nothing more, and let her ride and walk daily in the fresh air, and, my word for it, she shall have the rose in her cheeks, and the cherry on her lips, and the bound of joy in her step in less than six months. But, doctor, she can never live on such a plain diet. Why, Madam, there are at this moment tribes of Arabs who subsist for a whole life-time solely on milk, and that life-time is often one hundred years. And these clans have brains and soul and sentiment as well as brawn. Nature provides only one article of food for us when we start in life, and this appears to me a broad hint for a simple diet after we leave our mother's bosom. I once indulged, says Marmontel, in living for six weeks on milk only, while in full health. Never was my soul more calm, more peaceful. My days flowed along in study, with an unalterable equality. My nights were one gentle sleep. Discord might have overturned the world, it would not have shaken me.

To the children of Israel, who were to be strong and of good courage, to conquer their inheritance, fed from heaven day by day for forty years, God gave only one food. Do not despise my principle that the stomach is the mother of the life and the glow of lasting beauty. Love your stomach rather than be given to appetite. Give it perfect rest between meals. Above all, eat no night meals. They are the most flagrant violations of the law of rest for the stomach. They give, indeed, a flush, a glow, but it is a partial fever, a sign of nature's struggle, and in the morning you will come down with a pale face, the *bouche pateuse*, or clammy mouth, and you will want some sharp sauce to quicken appetite for breakfast, and your cup of coffee to wind up your spirit to a cheerful mood. All the disorders of the blood, palpitations and chest pains, gout, rheumatism and neuralgia, coldness of the extremities, pallor, headaches, sallowness, have their origin in the indigestions or maldigestions of our food. The liver belongs to the organs of digestion, and much as it is blamed by the doctors and the people for its sins, very rarely is it in fault unless the stomach be first abused and weakened.

After a good digestion, the next positive and unfailing specific for beauty is daily and moderate exercise in the open air. If it be the business of the stomach to make the blood of life good and pure at the fountain, it is the work of the breathing organs to keep it pure. Every three minutes all the blood of the body passes through the lungs, and the blood comes there to be changed from a dark and turbid current, which creeps slowly to the heart, into the beautiful crimson current, which bounds away on wings of life to every part of the body. How necessary is breath to life judge when I tell you that if the breath be stopped even partially as in asthma, agony is the only name for the suffering, and a perfect arrest of breathing, as in drowning, for five minutes, is fatal. Moses has told us the blood is the life of the flesh, and this is beautifully true, but only true of the blood after it has felt the breath of life in the lungs, and comes forth in crimson purity. The amount of breathing is nearly doubled in walking to what it is in sitting, and a fourth more in riding. Now, if the blood, before it touches the air in the lungs, is so full of the poisonous carbonic acid that it would kill in five minutes, then if we breathe less than we ought, by so much do we leave poison in the blood, and this robs the cheek of its glow and impairs all the powers and actions of life. There is a mysterious charm in *life*, in the animus and spirits of health, and these all belong to a true and inspiring beauty. All disease is a beginning, a tint, or hint, a shade of death, and these are begun in the stomach or begotten for want of more health. A manufacturer in France, who employed a great number of operatives, applied to a distinguished architect to have his factory carefully and evenly ventilated, and the demand for medicines in that establishment, to meet the ails of the operatives, went down from five thousand francs to less than five hundred francs in a single year.

Another of the specifics in the regime of beauty, is the salt friction bath. This should be taken in a warm room, with water at 85 degrees, and salt should be added, a tablespoonful to a half gallon of water. This should be a hand bath, rubbing a part of the body at a time, with the hands dipped in the water, in the same manner as the face is washed, and so go over the body, part by part, in turn. Do not uncover any part of the body before you are ready to give it the hand bath. Follow the bathing of each part by wiping with a linen towel, a *soft* one, and then follow by a *soft* flannel towel. Then cover that part of the body, and uncover and bathe the next in order. After a good digestion, and a pure blood from breathing much fresh air, daily, nothing more promotes a firm skin and a fine color than the salt friction, and given as I have advised, it may be taken daily. Thus given, it removes all impurities from the skin, is a tonic to the frame, and adds light and lustre and polish and purity to the complexion and to the entire body.

How vain is the effort, everywhere so common, while neglecting the natural laws of health, and the true sources of beauty, to attempt to patch up fading charms by the use of the poisonous cosmetics, lotions and enamels for the skin. A distinguished chemist has analysed seventeen of these compounds—solemn cheats, straws at which so many clutch, in the vain hope they can do what they promise—and he has found them to contain large quantities of lead and mercurial poisons.

All true beauty comes from within, from a pure and vigorous body, animated by a pure and vigorous soul. It is the glory of the hidden life, that shines through and makes the outer beautiful. A selfish heart disturbs the finest face. Bad passions spoil the blood and stamp ugliness on the finest lineaments. Badness and beauty cannot keep company. How often have we seen a truly beautiful face spoiled by vanity, by envy, by low thoughts and low associations. These passions strike through, and reveal their ugliness in the eye, in the lip, in the lines and wrinkles of the face. A lady, whose face was badly pitted by small-pox, told me her face was her first thought whenever she saw a stranger; and I said to myself, thus it is with bad natures, their first thought, as they come into the presence of others, is of their own evil heart, and this destroys the frankness and sweetness of an innocent spirit. All things are lost in aspirations for beauty, unless we fill the mind with great thoughts and lofty aims. We must have a purpose in life, and that purpose must be holy, and pure, and good, and absorbing. We must from our hearts forgive, and so forgive that we do not remember the offence, nor is it any more our first thought when we meet the offender. Thus God forgives us, and when we are forgiven of God, and all others wherein we have offended, and when last but not least we have truly forgiven ourselves, then we are at *rest,* and rest brings peace. There is no remedy for deformity of spirit or face but *love.* Even a naturally ugly face is transformed by it into one we love. Love is the great reformer, the great transformer. Nothing works good unsavored by *love.* God is love, and out of Zion the perfection of beauty, God hath shined. Let the *beauty* of our God be upon us.

